# Dans le sillage d'Alphonse Laveran

**DOI:** 10.48327/mtsi.v3i2.2023.331

**Published:** 2023-04-25

**Authors:** Patrick BERCHE

**Affiliations:** Professeur émérite, Université Paris Cité, 85 boulevard Saint-Germain 75006, Paris, France; * Actes du Colloque – Centenaire de la mort d'Alphonse Laveran. 24 novembre 2022, Paris / Proceedings of the Conference – Centenary of the death of Alphonse Laveran. 24 November 2022, Paris

**Keywords:** Alphonse Laveran, Malaria, Paludisme, Anophèles, Hématozoaire, Protozoaire, Artémisinine, Histoire de médecine, Alphonse Laveran, Malaria, Anopheles, Haematozoa, Protozoa, Artemisinin, History of medicine

## Abstract

Alphonse Laveran (prix Nobel 1907) a joué un rôle pionnier en découvrant l'agent responsable du paludisme, une maladie qui existe depuis des temps immémoriaux, et longtemps emblématique de la théorie des miasmes jusqu’à la fin du XIX^e^ siècle. En 1880, ce médecin militaire inconnu a mis au jour le rôle d'un hématozoaire dans la malaria, désigné *Plasmodium.* C’était le premier protozoaire dont le rôle était découvert dans une maladie infectieuse, à une époque où les bactéries étaient principalement soupçonnées. Cette découverte majeure a entraîné dans son sillage la mise en évidence du rôle des moustiques dans la propagation de la malaria par Ronald Ross (prix Nobel 1902) et Battista Grassi. La récurrence des accès palustres pendant des années a longtemps été une énigme qui ne fut résolue qu'après la Seconde Guerre mondiale par la découverte du cycle exo-érythrocytaire des *Plasmodium.* Les progrès ont ensuite concerné le traitement, allant de l’écorce de quinquina, la quinine, la chloroquine, jusque récemment l'artémisinine découverte en 1972 par la chercheuse chinoise Tu Youyou (prix Nobel 2015).

## Abstract (en)

Alphonse Laveran (Nobel Prize 1907) played a pioneering role in discovering the causative agent of malaria, a disease that has existed since time immemorial, and long emblematic of the miasma theory until the end of the 19^th^ century. In 1880, this unknown military doctor discovered the role of a hematazoan in malaria, designated *Plasmodium.* This was the first protozoan to be discovered in an infectious disease, at a time when bacteria were mainly suspected. This major discovery led to the identification of the role of mosquitoes in the spread of malaria by Ronald Ross (Nobel Prize 1902) and Battista Grassi. The recurrence of malaria attacks over many years was for a long time an enigma only solved after the Second World War by the discovery of the exo-erythrocytic cycle of *Plasmodium.* Progress was then made in treatment, from cinchona bark, quinine and chloroquine, to the recent discovery of artemisinin in 1972 by the Chinese researcher Tu Youyou (Nobel Prize 2015).

Le paludisme est une maladie humaine qui existerait depuis le Pléistocène. Au Néolithique, sa propagation est favorisée par le réchauffement climatique, l'agriculture et la croissance démographique. On trouve des descriptions de fièvres intermittentes dans des textes médicaux chinois, indiens, assyriens et grecs de l'Antiquité. Venu d'Afrique, le paludisme s'est répandu en Grèce à partir de l’Égypte pharaonique, comme en atteste la présence de traces biologiques des parasites sur de nombreuses momies égyptiennes. Hippocrate (v^e^ siècle av. J.-C.) décrit les fièvres de la fin de l’été (*febris tertiana, quartana, quotidiana, continua, tropica*). Les fièvres tierces sont parfois mortelles lors d'accès pernicieux avec coma terminal [9]. Pendant la guerre du Péloponnèse, la malaria aurait été responsable d'une grave épidémie qui a frappé l'armée athénienne lors du siège de Syracuse en Sicile, cité ceinte de marécages. Hippocrate associe la malaria aux miasmes émanant des marais. Pour l'auteur latin Varron (50 av. J.-C.), les marais sont infestés par des animaux minuscules qui causent les fièvres. Il faut donc éviter de respirer les vapeurs nauséabondes (*mal'aria,* mauvais air) des eaux stagnantes près de Rome.

La malaria se répand dans l'Empire romain et s'implante dans le sud de l'Europe, principalement en Italie [2]. Celse et Galien décrivent des fièvres rémittentes qu'ils associent à certaines conditions climatiques et environnementales (marais). Ces fièvres intermittentes ont aussi été rapportées par Archigène d'Apamée au II^e^ siècle et Aétius d'Amida au vi^e^ siècle. Au Moyen Âge, le paludisme s’étend vers le nord de l'Europe dans les contrées marécageuses le long des vallées de l'Elbe, du Rhin, du Rhône, du Pô et du Tibre. Le paludisme sévit aussi par exemple lors de la construction du château de Versailles en zone marécageuse. La maladie régresse au xviii^e^ siècle dans toute l'Europe du fait de l'assèchement des marais et de la régulation des cours d'eau. À cette époque, on dispose d'un traitement efficace, le premier contre des parasites, l’écorce de quinquina importée du Pérou par les jésuites et introduite en Europe au xvii^e^ siècle. Le paludisme était inconnu dans l'Amérique précolombienne, absent des traités de médecine des Mayas et des Aztèques. Il a été apporté par la traite des esclaves africains impaludés. On a observé de nombreuses épidémies très meurtrières sur le continent américain. Par exemple, la malaria a atteint 50 à 80 % des soldats lors de la guerre de Sécession entre 1861 et 1865. En 1914, on estime encore le nombre de cas de paludisme à plus de 600 000 aux États-Unis, où la maladie sévira jusqu’à la Seconde Guerre mondiale.

Le parasite protozoaire responsable du paludisme a été identifié par Alphonse Laveran (1845-1922) en 1880, et son mode de transmission par les moustiques anophèles par Ronald Ross (1857-1932) et Battista Grassi (1854-1925) en 1898. Ces découvertes sont à la base de la lutte contre la pandémie mondiale qui sévit depuis des siècles.

## La Découverte D'alphonse Laveran en 1880

Le médecin militaire Alphonse Laveran prend ses fonctions à l'hôpital militaire de Bône en Algérie en septembre 1878, puis il est détaché quelques mois à l'hôpital de Biskra à 300 km dans l'Atlas, avant d’être muté à l'hôpital de Constantine à partir de 1880. Il sera amené à prendre en charge de nombreux patients impaludés. À l'autopsie de malades morts de fièvre pernicieuse, il observe la présence de pigments noirs dans la rate, le foie et le cerveau. Il décide de rechercher ces pigments dans le sang de patients impaludés prélevé au doigt. Le sang frais est examiné au microscope entre lame et lamelle à un grossissement de 400 fois, sans coloration, à la lumière du jour! Malgré ces difficultés, il repère des leucocytes chargés de granulations de mélanine, les « leucocytes mélanifères ». Il découvre ensuite ces pigments dans des globules rouges déformés, souvent en croissant, ou sphériques pourvus de filaments périphériques avec des mouvements amiboïdes, ou encore sphériques déformés et sans filaments. Laveran écrira plus tard en parlant de sa découverte :

« En étudiant dans le sang frais des malades atteints de fièvre palustre les éléments pigmentés, je remarquai qu’à côté des leucocytes mélanifères, on trouvait des éléments de forme assez régulière (corps sphériques et corps en croissant) bien différents des leucocytes; le 6 novembre 1880 je constatai, à Constantine, dans le sang d'un malade, l'existence de corps sphériques pigmentés, de corps en croissant et de flagelles très mobiles; dès lors je n'eus plus de doutes sur la nature animée des éléments qui, depuis quelque temps, avaient attiré mon attention et je décrivis les trois formes principales sous lesquelles se présente l'hématozoaire du paludisme : corps amiboïdes, corps en croissant, flagelles [16]. »

Le sulfate de quinine fait disparaître ces anomalies des érythrocytes. Les corps en croissant (en réalité des gamétocytes) sont retrouvés chez 148 patients, mais chez aucun des 52 patients hospitalisés indemnes de malaria. Il remarque aussi que les Européens ont un sang beaucoup plus riche en parasites que les autochtones, ce qu'il met en rapport avec « la force de résistance que ces hommes présentent à l'impaludisme ». Les filaments mobiles ayant des analogies avec des oscillariées (algues), il les nomme *Oscillaria malariae.* C'est la première fois qu'on identifie un protozoaire à l'origine d'une maladie infectieuse.

Convaincu que ce parasite protozoaire des globules rouges est l'agent du paludisme, il envoie une note de 8 pages [13] à Léon Colin, médecin militaire récemment élu à l'Académie de médecine, pour présenter ses travaux en séance. Laveran joint aussi ses observations d'hématozoaires proches chez les oiseaux [14]. L'académicien communique ces résultats les 23 novembre et 28 décembre 1880, de façon très succincte et avec des réserves. Cette découverte fut reçue avec indifférence et incrédulité, considérant qu'il s'agissait d'artefacts. À l’époque, le paludisme est une des maladies emblématiques, avec la fièvre jaune, de la théorie des miasmes d'Hippocrate, encore défendue par beaucoup de médecins. Peu de temps avant la découverte de Laveran, Robert Koch avait montré en 1876 le rôle d'une bactérie lors d'une épidémie bovine de charbon et Louis Pasteur venait de présenter à l'Académie de médecine sa célèbre « théorie des germes » en 1878. Les nouveaux partisans du courant contagioniste de Pasteur et Koch étaient alors centrés sur une transmission des germes contagieux par inhalation ou par ingestion d'eau. C'est ainsi qu'en 1879, deux éminents bactériologistes, Edwin Klebs et Corrado Tommasi-Crudeli, réussissent à isoler en culture à partir de prélèvements d'eau des marais Pontins, région de haute endémie palustre au sud de Rome, un bacille qu'ils nomment *Bacillus malariae.* Injectée à des lapins, cette bactérie induit une forte fièvre avec splénomégalie, mimant en apparence des signes de malaria [11].

Comment accepter qu'un officier français inconnu travaillant sur le terrain en Algérie puisse mettre en cause le dogme de l'antique théorie des miasmes et celui de l'origine bactérienne de la malaria ! Ce fut pour Laveran une traversée du désert jusqu’à son retour en France en 1884 avant qu'on ne reconnaisse l'immense valeur de ces observations. Habité par la conviction en sa découverte, il va s'acharner à convaincre. Laveran rassemble en 1884 l'ensemble de ses travaux cliniques et biologiques dans un *Traité des fièvres palustres* [15]. Il y mentionne son échec à cultiver le parasite à partir du sang des malades, de l'eau et du sol de localités insalubres, et son incapacité de reproduire un paludisme expérimental chez le lapin. Il émet aussi l'hypothèse que les moustiques abondants dans toutes les zones palustres pourraient jouer un rôle important dans la propagation du paludisme.

En 1885, les chercheurs italiens spécialistes reconnus du paludisme, Ettore Marchiafava (1847-1935) et Angelo Celli (1857-1914) confirment les observations de Laveran [20]. Ils renomment l'hématozoaire *Plasmodium.* Conjointement, Pasteur, Chamberland et Roux valident sa découverte. Malgré cela, la théorie des miasmes à l'origine de la malaria persistera jusqu'en 1895. Laveran recevra le prix Nobel en 1907. Il écrira : « Les découvertes exactes sont quelquefois accueillies avec un scepticisme absurde parce qu'elles ne cadrent pas avec les idées reçues, pendant que les pseudo-découvertes qui s'accordent avec les opinions courantes sont acceptées de tous, bien qu'elles reposent sur un très petit nombre de preuves. » La découverte de l'agent du paludisme en 1880 par Laveran a été à l'origine de nombreuses recherches sur un des fléaux les plus mortels de l'Humanité.

## Le Rôle Des Moustiques Par Ronald Ross en 1898

L'idée que des germes puissent être transmis par des vecteurs animaux a été mise au jour par un naturaliste russe, Aleksei Fedchenko (1844-1873), qui a montré lors d'un voyage au Turkestan le rôle du *Cyclops,* minuscule crustacé aquatique, dans la transmission du ver de Guinée à l'origine de la filaire de Médine. En effet, il avait observé au microscope la présence de nombreuses larves dans ces copépodes qui contaminent l'eau de boisson et transmettent ainsi la filariose [3]. Cette idée d'un vecteur permettant la transmission d'un parasite a été confortée par les travaux de Patrick Manson (1844-1922) qui a démontré en 1878 le rôle des moustiques du genre *Culex* dans la transmission des microfilaires présentes dans le sang des patients souffrant d’éléphantiasis [18]. Reprenant les idées de Laveran, Manson publie en 1894 une théorie sur la propagation du paludisme par les moustiques [19].

C'est Ronald Ross, un jeune médecin militaire britannique de l'Indian Medical Service, qui va résoudre l’énigme de la transmission du paludisme. Entré au service de Sa Majesté en 1881, il est envoyé en Extrême-Orient en 1890 et s'intéresse à partir de 1892 à la malaria, cause majeure des fièvres qui frappent près d'un tiers des 300 000 soldats de l'Armée des Indes. Il connaît les travaux de Laveran et en 1894 il rencontre à Londres Manson qui l'incitera à tester l'hypothèse d'une transmission par les moustiques de la malaria. Il correspondra avec lui pendant 4 ans [24]. De retour en Inde en avril 1895, il est envoyé à Madras en zone de forte endémie palustre. Bien que n'ayant aucune notion d'entomologie, il examine au microscope des milliers de moustiques de toutes sortes d'espèces, exposés à des patients impaludés. En juin 1897, il est muté à Secunderabad. C'est là qu'après 2 ans d'efforts infructueux, il réussit en juillet 1897 à cultiver 20 moustiques bruns adultes à ailes tachetées (que l'on sait aujourd'hui être des anophèles), qu'il expose à des patients impaludés. Quelques jours plus tard, il observe le 20 août 1897 des « spores » pigmentées (oocystes) dans la paroi intestinale des moustiques qui libèrent des bâtonnets (sporozoïtes). Il publie sa découverte le 27 août 1897 dans l’*Indian Medical Gazette* [26], puis dans le *British Medical Journal* en décembre 1897 [27]. Ses recherches sont alors interrompues par sa mutation à Bombay en septembre 1897 puis dans le Rajputana (Rajasthan), une zone sans paludisme où il se morfond. Muté en février 1898 à Calcutta pour travailler au Presidency General Hospital, il se résigne à étudier, sur le conseil de Manson, le paludisme des oiseaux dû à une espèce proche de parasite, *Plasmodium relictum.* En juillet 1898, il observe des « spores » pigmentées (oocystes) dans la paroi intestinale des « moustiques gris » (*Culex fatigans*) nourris avec des oiseaux infectés (178 positifs sur 242 examinés) : les gamétocytes du sang produisent des oocystes à la surface de l'intestin. Il démontre le rôle des « moustiques gris » dans la malaria aviaire et observe le 4 juillet que les glandes salivaires des moustiques sont le site de stockage des parasites en bâtonnets qui sont inoculés par la salive des moustiques femelles lors des piqûres. Il réussit aussi à transmettre les parasites par les moustiques infectés à des moineaux indemnes [28]. En 1899, il reproduit en Sierra Leone ses observations chez des patients impaludés piqués par des anophèles. Dans le premier numéro des *Annales de l'Institut Pasteur* de l'année 1899, Ross publie un article daté du 31 décembre 1898 intitulé « Du rôle des moustiques dans le paludisme » où il incrimine des moustiques « gris à ailes tachetées » qui s'avèreront être des anophèles [25]. Cette découverte du rôle des moustiques dans la transmission du paludisme lui vaudra le prix Nobel de médecine en 1902, « pour ses travaux sur le paludisme, par lesquels il a montré comment il pénètre dans l'organisme ».

## Battista Grassi et L’école Italienne

Laveran pensait que le parasite du paludisme était polymorphe mais n'appartenait qu’à une seule espèce et que les fièvres tierce ou quarte ne dépendaient pas des formes parasitaires sanguines. Les chercheurs italiens, qui avaient une longue expérience du paludisme très répandu à l’époque dans leur pays, ont montré qu'en réalité il existait plusieurs espèces d'hématozoaires en fonction des fièvres palustres. En 1889, Camillo Golgi (1843-1926) observe des morphologies différentes entre les *Plasmodium* de la tierce bénigne et de la tierce maligne [5]. En 1890, Battista Grassi, médecin et zoologiste, et Amico Bignani (1862-1929) distinguent *P. vivax* (tierce bénigne), *P. falciparum* (tierce maligne) et *P. malariae* (quarte). Dès 1896, Grassi et Bignani suspectent les piqûres de moustiques dans la transmission des *Plasmodium,* sans transmission trans-ovarienne. En 1898, Grassi, Bignani et Giuseppe Bastianelli (1862-1959) réussissent la première transmission expérimentale de *P. vivax* à l'homme par un moustique, *Anopheles claviger,* à l'hôpital San Spirito de Rome [6]. En 1899, les mêmes auteurs décrivent le cycle de développement de *P. vivax, P. falciparum et P. malariae* chez l'anophèle dans le corps de *A. claviger* [1, 8]. La transmission par les anophèles chez l'homme sera confirmée par une expérience réalisée dans la région d'Ostie par des chercheurs britanniques. Près de 112 volontaires protégés la nuit en zone d'endémie demeurèrent indemnes de paludisme, alors que 415 volontaires non protégés furent tous impaludés [29]. Grassi a résumé l'ensemble de ses travaux en 1900 [7]. Ainsi faut-il souligner l'apport considérable des chercheurs italiens, notamment Battista Grassi non récompensé du prix Nobel, dans la découverte du rôle d*‘Anopheles claviger* (parmi les 55 espèces d'anophèles existant en Italie) dans le paludisme humain, celle du cycle de vie des *Plasmodium* dans les globules rouges et les moustiques, et la description des différentes espèces de *Plasmodium.*

## DéCouverte du Cycle Exo-érythrocytaire Des Plasmodium

Le cycle érythrocytaire des *Plasmodium* dans le sang et le cycle sporogonique dans les anophèles étaient bien décrits dès le début du xx^e^ siècle. Mais il restait plusieurs questions non résolues. Comment expliquer le délai de 10 à 14 jours entre la piqûre du moustique et l'apparition des parasites dans le sang ? Comment expliquer les rechutes pendant des années, notamment avec *P. vivax*? En 1903, le zoologiste Fritz Schaudinn publie des dessins montrant la pénétration directe de sporozoïtes de *P. vivax* dans des hématies. L'invisibilité des hématozoaires serait due à leur faible nombre. Nul besoin d'un gîte extra-érythrocytaire [30]. Cette observation (non reproductible) faite par un éminent chercheur allemand va faire perdre des décennies à la réelle explication de la disparition des parasites dans le sang.

Dès 1898, le Canadien William George MacCallum (1874-1944) avait mis au jour une phase de multiplication des parasites dans le système réticulo-endothélial de la moelle, du foie et de la rate au cours du paludisme aviaire à *P. relictum* [17]. Dans son ouvrage sur le paludisme publié en 1926, le pasteurien Émile Marchoux évoque trois mécanismes de rechute du paludisme humain : la réactivation d'une forme sanguine enkystée; une parasitémie *a minima* associée à une immunité suffisante pour la contrôler mais insuffisante pour l’éliminer; une parthénogenèse des gamétocytes présents dans le sang de manière continue, mais en nombre trop faible pour être détectés [21]. En 1934, G. Raffaelle découvre la présence de *P. elongatum* des oiseaux dans les cellules de la moelle osseuse, puis de *P. relictum* dans des cellules réticulo-endothéliales de la moelle, du foie et de la rate. Pendant la période de latence, le sang des oiseaux n'est pas infectieux [23]. En 1937, Sydney James et P. Tate retrouvent les parasites (*P. gallinaceum*) dans le système réticulo-endothélial et les cellules endothéliales des capillaires cérébraux des poulets [10]. Ces résultats étaient acceptés avec l'idée qu'ils demeuraient spécifiques des oiseaux et ne concernaient pas les mammifères. Cependant, au décours de la Première Guerre mondiale s’était développée la malariothérapie à *P. vivax* pour traiter la neurosyphilis, sous l'impulsion du psychiatre Julius Wagner von Jauregg (1857-1940), prix Nobel 1927 [35]. Certaines observations cliniques étaient en faveur d'un cycle exo-érythrocytaire des *Plasmodium.* D'une part les rechutes survenaient après une exposition aux moustiques impaludés (injectant des sporozoïtes), mais pas après une inoculation du sang de patients (contenant uniquement des formes asexuées). D'autre part, le traitement par la quinine pendant l'incubation ne prévenait pas l'apparition des accès si on utilisait une inoculation par les moustiques.

Au seuil de la Seconde Guerre mondiale, il était admis qu'il existait un cycle exo-érythrocytaire lors du paludisme aviaire. Ce qui était vrai pour les oiseaux le serait-il pour les mammifères ? Il existait quelques publications dans les années 1940 montrant la présence de parasites dans les tissus, notamment dans le cerveau des primates et de chauves-souris souffrant de malaria. En 1947, Henry Shortt (1887-1987) et Cyril Garnham (1901-1994) à Londres montrent que des singes (*P. cynomolgi*) exposés à des anophèles infectés présentent des parasites dans les hépatocytes du foie [4, 31], de même que des volontaires humains [32]. Les parasites se multiplient dans les hépatocytes du foie avant d’être présents dans le sang. Cela a mené à la découverte en 1982 des hypnozoïtes, une forme dormante dans les hépatocytes [12]. Les cycles exo-érythrocytaires dans le foie entretiennent la parasitose pendant 3 à 5 ans pour *P. vivax,* 2 à 3 ans pour *P. ovale,* et pendant la vie entière pour *P. malariae.*

## Origine Des Plasmodium

Les *Plasmodium* des mammifères, oiseaux et reptiles, proviendraient de protozoaires aquatiques qui auraient infecté des diptères (ancêtres des moustiques) il y a 150 à 200 millions d'années. Les études phylogéniques montrent que *P. falciparum* est très proche de *P. reicheinowi,* parasites du gorille et du chimpanzé. En revanche, *P. vivax, P. malariae* et *P. ovale* appartiennent à un clade différent, incluant tous les *Plasmodium* des autres primates. Certains parasites des singes (*P. cynomolgi, P. schwetzi, P. simium*) ont divergé de la lignée de *P. vivax.* On sait aujourd'hui que les *Plasmodium* humains sont originaires d'Afrique et se sont répandus lors des migrations des premiers hominidés hors d'Afrique.

La longue cohabitation entre les parasites du paludisme et l'homme est attestée par la sélection de mutations humaines protectrices exprimées dans les érythrocytes, notamment impliquant les hémoglobines [34]. Alors que les hétérozygotes sont plus résistants au paludisme, le tribut à payer est celui des homozygotes qui développent des maladies souvent mortelles. En Afrique, on relève la thalassémie (HbA, HbA2, HbF), la drépanocytose (HbS), le déficit en G6PD et les érythrocytes Duffy-négatif; en Asie la thalassémie et les hémoglobinopathies (Hb C, E); et en Mélanésie les ovalocytoses (mutation d'une protéine de transport).

## Le Traitement du Paludisme

On a rapporté d'Amérique l’écorce sacrée du quinquina, arbre de l'Altiplano, connu des Indiens d'Amérique du Sud pour son efficacité sur les fièvres. Au xvii^e^ siècle, les jésuites détiennent le monopole du très lucratif commerce de la poudre des jésuites (ou poudre de la comtesse). L’écorce de quinquina est importée en Espagne dès 1630, puis en Angleterre en 1679 par le pharmacien Robert Talbot et Thomas Sydenham. En 1820, les pharmaciens Pierre-Joseph Pelletier (1788-1842) et Joseph Bienaimé Caventou (1795-1877) en extraient des alcaloïdes actifs sur les fièvres, la quinine et la cinchonine, qu'ils fabriquent industriellement dès 1824. En 1934, la firme Bayer met au point le premier dérivé synthétique de la quinine : la chloroquine. Devant l'apparition progressive des résistances aux antipaludéens, une importante recherche est entreprise, aboutissant à la découverte de l'artémisinine en 1972. En 1967, un projet dit 523 est développé en Chine pour rechercher de nouveaux antipaludiques permettant de lutter contre la résistance des *Plasmodium* à la chloroquine. En 1969, Tu Youyou étudie les herbes de la pharmacopée traditionnelle chinoise et recueille 2000 recettes de médecine traditionnelle. Elle prépare et teste 300 extraits d'herbes. Un composé dérivé *d'Artemisia annua,* connue pour être active sur les fièvres intermittentes (décrite dans un texte chinois du iii^e^ siècle), s'avère efficace sur le paludisme expérimental [22]. En 1972, le Pr Tu avec son équipe isole l'artémisinine dont elle décrit la structure chimique, la pharmacologie et l'efficacité sur le paludisme. En 1981, elle présente ses résultats lors d'un congrès de l'OMS [33]. L'exploit est d'autant plus impressionnant que près de 240 000 composés synthétiques ont été préparés et testés par les scientifiques du monde entier, sans succès. Le Pr Tu Youyou a reçu le prix Nobel de médecine en 2015.

En conclusion, le paludisme demeure un fléau endémique mondial responsable d'une forte mortalité infantile en Afrique et en Asie, malgré un recul progressif depuis la fin de la Seconde Guerre mondiale. D'après le rapport de l'OMS de 2021, il y a eu dans le monde 241 millions de cas en 2020 avec un accroissement du nombre de *P. falciparum* et une diminution de *P. vivax.* La mortalité annuelle est estimée à 677 000 décès. Il existe des résistances à l'artémisinine en Asie qui émergent également en Afrique. La malaria reste un problème majeur sanitaire et socio-économique dans beaucoup de pays, notamment en Afrique subsaharienne.

## Liens D'intérêts

L'auteur ne déclare aucun lien d'intérêt.
